# Antibody against apolipoprotein-A1, non-alcoholic fatty liver disease and cardiovascular risk: a translational study

**DOI:** 10.1186/s12967-023-04569-7

**Published:** 2023-10-05

**Authors:** Sabrina Pagano, Stephan J. L. Bakker, Catherine Juillard, Stefania Vossio, Dimitri Moreau, Karim J. Brandt, François Mach, Robin P. F. Dullaart, Nicolas Vuilleumier

**Affiliations:** 1 Division of Laboratory Medicine, Diagnostics Department, Geneva University Hospitals, Rue Michel Servet 1, 1211 Geneva, Switzerland; 2 Department of Medicine Specialties, Medical Faculty, Geneva University, Geneva, Switzerland; 3 Department of Internal Medicine, Division of Nephrology, University of Groningen, University Medical Center Groningen, Groningen, The Netherlands; 4 School of Chemistry and Biochemistry, National Centre of Competence in Research (NCCR) Chemical Biology, University of Geneva, Geneva, Switzerland; 5 Department of Cardiology, University Hospitals of Geneva, Geneva, Switzerland; 6 Department of Internal Medicine, Division of Endocrinology, University Medical Center Groningen, University of Groningen, Groningen, The Netherlands

**Keywords:** Anti-apolipoprotein A-1 antibodies, NAFLD, Triglyceride pathway, Inflammation, Cytokeratin-18, Cardiovascular risk, Framingham Risk Score (FRS)

## Abstract

**Background:**

Non-alcoholic fatty liver disease (NAFLD) is a common liver disease increasing cardiovascular disease (CVD) morbidity and mortality. Autoantibodies against apolipoprotein A-1 (AAA-1) are a possible novel CVD risk factor promoting inflammation and disrupting cellular lipid homeostasis, two prominent pathogenic features of NAFLD. We explored the role of AAA-1 in NAFLD and their association with CVD risk.

**Methods:**

HepaRG cells and liver sections from ApoE−/− mice exposed to AAA-1 were used for lipid quantification and conditional protein expression. Randomly selected sera from 312 subjects of the Prevention of Renal and Vascular End-stage Disease (PREVEND) general population cohort were used to measure AAA-1. A Fatty Liver Index (FLI) ≥ 60 and a 10-year Framingham Risk Score (FRS) ≥ 20% were used as proxy of NAFLD and high CVD risk, respectively.

**Results:**

In-vitro and mouse models showed that AAA-1 increased triglyceride synthesis leading to steatosis, and promoted inflammation and hepatocyte injury. In the 112 PREVEND participants with FLI ≥ 60, AAA-1 were associated with higher FRS, alkaline phosphatase levels, lower HDL cholesterol and tended to display higher FLI values. Univariate linear and logistic regression analyses (LRA) confirmed significant associations between AAA-1, FLI and FRS ≥ 20%, while in adjusted LRA, FLI was the sole independent predictor of FRS ≥ 20% (OR: 1.05, 95%CI 1.01–1.09, P = 0.003). AAA-1 was not an independent FLI predictor.

**Conclusions:**

AAA-1 induce a NAFLD-compatible phenotype in vitro and in mice. Intricate associations exist between AAA-1, CVD risk and FLI in the general population. Further work is required to refine the role of AAA-1 in NAFLD and to determine if the AAA-1 association with CVD is affected by hepatic steatosis.

**Supplementary Information:**

The online version contains supplementary material available at 10.1186/s12967-023-04569-7.

## Background

Non-alcoholic fatty liver disease (NAFLD) is defined by hepatic steatosis (triglycerides > 5.5% of liver volume) arising in the absence of significant alcohol intake and without evidence of injury or fibrosis [1]. The estimated annual medical costs directly attributable to NAFLD exceed thirty-five billion euros in Europe and one hundred billion dollars in the United States [1, 2].

With the gradual prevalence increase observed over the last years, one hundred million people are predicted to be affected by NAFLD worldwide by 2030, and NAFLD may become a major cause of cirrhosis and consequently, liver transplantation [1, 3]. As a systemic condition, NAFLD also carries a broad range of extra-hepatic complications, including cardiovascular diseases (CVD), type 2 diabetes (T2D) and other diseases [4].

Although obesity is a major preventable NAFLD risk factor, up to 20% of the cases occur in lean individuals where no other risk factors can be identified [5, 6]. The picture is further complicated by the fact that the definite diagnosis mostly relies upon histological features that are poorly accessible at a general population level [1], and thus NAFLD screening and diagnosis remain a medical challenge in terms of prevention, early diagnosis and treatment [4]. Several NAFLD risk stratification tools integrating demographic factors and biomarkers have been derived with some successes in the general population, such as the Fatty Liver Index (FLI), Fibrosis-4 (FIB-4), the NAFLD fibrosis score (NFS) or the Enhanced Liver Fibrosis (ELF) panel. However, optimal NAFLD risk stratification is still an unmet medical need, in part due to the lacunar understanding of NAFLD pathophysiology.

Increased hepatocyte lipogenesis in response to increased fatty acid (FA) uptake seems to play a key role in the cascade of events leading to hepatocytes steatosis, inflammation and fibrosis [7, 8]. Such accumulation exceeding FA oxidation and cellular export capacities induces a hepatic inflammatory response mediated, among other processes, by receptor-mediated uptake of oxidized lipids, further amplifying a self-perpetuating pathogenic feedback loop [7, 8]. Therefore, any compound interfering with intracellular lipid regulation and inflammation could theoretically represent a potential mediator of NAFLD. Supporting this, autoantibodies of various specificities are frequently found in NAFLD [9, 10], including those against apolipoprotein B-containing oxidized lipids known to antagonistically modulate the course of macrophage foam cell formation [11].

Recently, the class of autoantibodies targeting apolipoprotein A-1 (AAA-1) has been experimentally demonstrated to interfere with macrophage lipid homeostasis promoting inflammation, and leading to foam cell formation [12–15]. Furthermore, they represent an independent cardiovascular (CV) risk factor in diverse populations, both in the general population and within various clinical contexts [12–14, 16–18]. In light of AAA-1’s antagonistic effects on crucial protective functions of apolipoprotein A-1 in NAFLD and metabolic syndrome [19], as well as their pro-inflammatory and pro-atherogenic properties, our study aimed to ascertain whether AAA-1 may play a role in mediating hepatic steatosis, inflammation, and hepatocyte injury. We also sought to investigate if these mechanisms could elucidate the association between NAFLD and heightened CV risk.

Using a translational approach combining in vitro and in vivo models with a general population cohort with FLI as a proxy of NAFLD diagnosis, we report an association between AAA-1, NAFLD, and the Framingham risk score (FRS) for the first time.

## Methods

### Cells

HepaRG cells [20], obtained from Biopredic International (Saint Gregoire, France) and were cultured as described in Additional file 1: Methods.

### In vivo study

Eleven-week old ApoE−/− mice were passively immunized with AAA-1 and the respective control IgG for 16 additional weeks, according to our previously described protocol [17, 18, 21].

Briefly, mice were fed under the standard chow diet and received intravenous endotoxin-free (< 0.25 EU/ml using the limulus amebocyte lysate endochrome assay) goat polyclonal AAA-1 or goat polyclonal IgG controls (50 µg per mouse per injection) every two weeks for 16 weeks as described in the previous study [17, 18, 21].

The investigation conforms to the Guide for the Care and Use of Laboratory Animals published by the US National Institutes of Health (NIH Publication No. 85-23, revised 1996) and was approved by the local and ethics authorities (Geneva Veterinary Office and the Ethic Commission of Animal Experimentation of the University of Geneva, 146/3710/2). This study conformed to the “position of the American Heart Association on Research Animal Use”. At the end of 16 weeks, the mice were euthanized, and their livers were collected.

Isolated liver tissues were embedded in paraffin. Seven-μm-thick liver tissue sections were taken and fixed with formalin (10%) (Sigma-Aldrich), and then subjected to histological analysis.

### Oil Red O (ORO) staining for lipid droplet distribution

ORO (Sigma-Aldrich), a fat-soluble dye, was used to detect neutral lipids (triglycerides, diacylglycerols, and cholesterol esters) in cryosectioned liver tissue. For details on methods and image acquisition, please refer to Additional file 1: Methods.

### Bodipy staining for lipid droplet distribution

Immunofluorescent detection of lipid droplets in HepaRG cells was done with the lipophilic dye Bodipy 493/503, 4,4-difluoro-3a,4a-diaza-s-indacene (Life Technologies, CA USA) while cell nuclei were stained with fluorescent stain Hoechst 33342 (Life Technologies). For further methodological and image acquisition details, please refer to Additional file 1: Methods.

### Protein purification and western blot analysis

Differentiated HepaRG cells were plated onto 6-well plates (Costar), at a density of 1.8 × 10^6^ cells per well. The day after, the cells were treated for 24 h with polyclonal AAA-1 (Academy Bio-Medical Co, ref: 11AG2) or control IgG (Meridian Life Science, ref: A66200H) at 37 °C at a concentration of 40 μg/mL. For details in protein extraction and western blot analysis, please refer to Additional file 1: Methods.

### Cytokines assessment

Supernatants derived from cells exposed to AAA-1 or control IgG for 24 h were analyzed for Interleukin 6 (IL-6), Interleukin 8 (IL-8), Tumor Necrosis Factor (TNF)-α, nd IL-10 secretion. Cytokines were measured using the V-Plex Proinflammatory Panel 1 kit from MesoScale Discovery (MSD) (Rockville, MD, USA) on the SQ120 instrument, following the manufacturer's instructions.

### siRNA-mediated SREBP-1 gene knock-down

siRNA transfections were performed with Lipofectamine RNAi-MAX (Invitrogen) in antibiotic-free medium according to manufacturer instructions. The detailed transfection protocol is described in Additional file 1: Methods.

### Blocking of TLR2/4 and CD14 receptors

HepaRG cells were treated for 30 min with blocking antibodies to TLR-2 (TL2.1 and TL2.5), TLR-4 and CD14 receptors (BioLegend; anti-TL2.1, ref: 309716; anti-TL2.5, ref: 121808; anti-TLR-4, ref: 315814; anti-CD14, ref: 325602) at a concentration of 20 μg/mL and followed by the addition of AAA-1 (40 μg/mL), or control IgG (40 μg/mL) for 24 h.

Supernatants were recovered and IL-6, IL-8 and TNF-α secretion was measured as mentioned in the paragraph of Cytokines assessment.

### The PREVEND general population cohort

The PREVEND (Prevention of Renal and Vascular Endstage Disease) cohort is a large population-based study including 8592 individuals aged 28–75 years from the city of Groningen (Netherlands) [22]. From these, 6066 participants completed the third screening PREVEND study round (2004–2007) where active infectious hepatitis and alcohol consumption have been excluded by a detailed questionnaire and for which an extensive clinical and biological characterization is available. For the purpose of the current study, we randomly selected 312 individuals with available fasting serum aliquots stored at − 80 °C for Fatty Liver Index (FLI) and AAA-1 assessment. The study was approved by the local ethical committee (Medisch Ethische Toetsingscommissie, abbreviated Metc, IRB no. 01/139) from the University of Groningen (Netherlands) and performed according to the Helsinki declaration. Further information on PREVEND cohort can be found at: https://research.rug.nl/en/datasets/prevention-of-renal-and-vascular-end-stage-disease-prevend.

### NAFLD definition in PREVEND

Suspected NAFLD was ascertained using the Fatty Liver Index (FLI) and defined as a FLI ≥ 60 as a validated proxy to detect NAFLD in the general population [23–25].

The FLI is calculated according to the following formula: FLI = (e0.953 * loge (triglycerides + 0.139 * BMI + 0.718 * loge (GGT) + 0.053 * waist circumference − 15.745)/(1 + e0.953 * loge (triglycerides) + 0.139 * BMI + 0.718 * loge(GGT + 0.053 * waist circumference − 15.745) * 100, where GGT is gamma-glutamyltransferase, and BMI is body mass index.

### CVD risk prediction assessment

Absolute risk for 10-year CVD was computed using the Framingham risk score (FRS) [26] algorithm, based upon gender, age, systolic blood pressure, treatment for hypertension, smoking, presence of diabetes, total cholesterol and HDL cholesterol [26]. According to latest recommendations, cardiovascular risk was considered as low if the FRS was less than 10%, moderate between 10 to 19%, and high if equal or above 20% [27].

### Biomarker determinations

Total cholesterol, HDL cholesterol and triglycerides (TG) were measured using routine procedures on a Roche Modular Platform chemistry analyzer (Roche 8000/H Cobas), low-density lipoprotein (LDL) cholesterol were calculated using the Friedewald formula. Glucose, Gamma-glutamyltransferase (GGT), alkaline phosphatase (ALP), alanine aminotransferase (ALT) and aspartate aminotransferase (AST) were quantified on a Roche Modular Platform.

### AAA-1 assessment

AAA-1 were measured as previously described [14, 16, 28]. Briefly, Maxisorp plates (NuncTM, Roskilde, Denmark) were coated with purified, human-derived delipidated and unmodified apoA-1 (20 µg/mL; 50 µL/well) for 1 h at 37 °C.

The detailed Elisa procedure is described in Additional file 1: Methods.

### Statistical analyses

Continuous variables were expressed as medians and interquartile ranges (IQR) while categorical variables were expressed in numbers with percentages. Normality of distribution was tested with the Shapiro–Wilk test. Comparisons between groups were performed using the non-parametric Mann–Whitney U test and Chi-square test when appropriate. Correlations analyses were carried out using Spearman rank correlation test. In linear regression analysis, non-normally distributed variables were transformed into a natural logarithmic value.

Univariate and adjusted logistic regression analyses were performed to determine the associations between AAA-1 seropositivity, a high 10-year CV risk according to FRS > 20% [27] and the risk of having NAFLD based upon a FLI score ≥ 60 [27]. Results are reported with 95% confidence intervals (95%CI) and IQR. Due to the predefined study endpoints and the exploratory nature of this work, adjustments for multiple testings were not performed. Statistical analyses were performed with Tibco Statistica software (version 13.5.0.17, TIBCO Software Inc., Palo Alto, CA, USA) on the PREVEND cohort, while GraphPad Prism 9.0.1 software (GraphPad Prism, CA USA) using the non-parametric unpaired Mann–Whitney U test was used for the in vitro an in vivo data or Kruskal–Wallis test was used for multiple comparisons. Statistical significance was set at p < 0.05.

## Results

### AAA-1 promote hepatic steatosis in vitro and in vivo

**Fig. 1:**
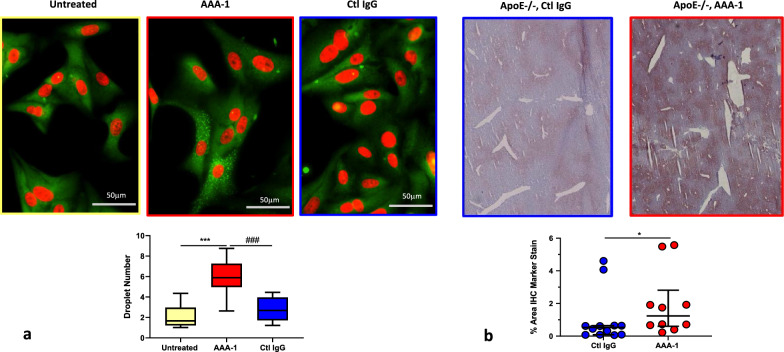
AAA-1 increase lipid droplet content in in vitro and in vivo. **a** Lipid droplet quantification is expressed as median with IQR and range of three independent experiments (N = 3). 800–1000 cells are analysed for each condition. *P*-value calculated with Mann–Whitney test. *** *P* = 0.001, ### *P* = 0.001. **b** ApoE−/− mice were immunized for 16 weeks. Lipid content in mice liver sections was evidenced using Oil Red O staining. Results are expressed as individual values and median with range. There were twelve mice for the control IgG treated group and ten mice for the AAA-1 treated group. *P* = 0.030 by Mann–Whitney test

As shown previously on macrophages [12, 14] our results, which are summarized in Fig. 1a, indicate that 24 h of AAA-1 exposure induced a significant increase in lipid droplet content in hepatic HepaRG cells, while no effect was observed in untreated or control IgG-treated cells. In an attempt to reproduce this phenotype in vivo, we applied our validated AAA-1 passive immunization protocol to apolipoprotein E knockout (ApoE-/-) mice [17, 18, 21].

As shown in Fig. 1b, after 16 weeks of passive immunization ApoE−/− AAA-1 recipient mice had higher hepatic lipid content upon histological examination when compared to control IgG treated mice.

Together, these results indicate that AAA-1 could promote hepatic steatosis either by increasing lipogenesis, promoting fatty acid uptake, or decreasing triglyceride oxidation or efflux via very low density lipoprotein (VLDL) secretion [7].

### AAA-1 affect the triglyceride pathway

Since triglycerides are the main neutral lipids present in hepatocyte lipid droplets [29], and the main lipids that accumulate in NAFLD, due to uncontrolled lipogenesis [7], we looked at the impact of AAA-1 on the lipogenesis pathway [30]. We therefore evaluated by Western Blot analysis whether AAA-1 could modulate the sterol regulatory element binding protein (SREBP)-1, one of the master transcription factors that controls the triglyceride pathway [31]. We also analyzed the expression of SREBP-2, which controls the cholesterol pathway [31] and was previously found to be upregulated in macrophages exposed to AAA-1 [12]. In addition, we looked for possible interference caused by these autoantibodies on: (i) the fatty acid synthase (FASN) enzyme catalyzing fatty acids synthesis, and (ii) the glycerol-3-phosphate acyltransferase (GPAT1) as the rate-limiting enzyme in the pathway of triglyceride synthesis required for fatty acid esterification [31].

**Fig. 2:**
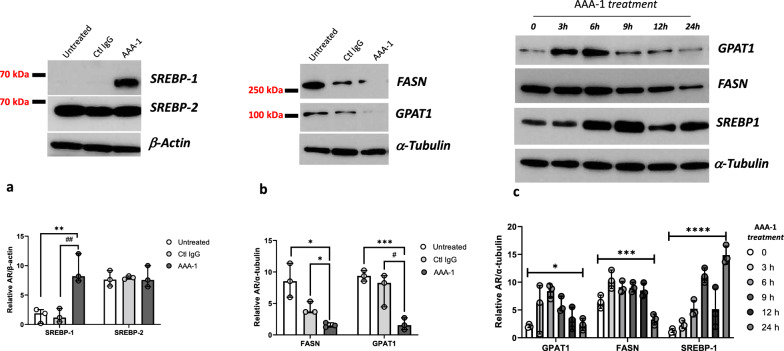
AAA-1 affect triglyceride metabolism. Western Blot assay was performed using HepaRG cells lysates, untreated, treated with Ctrl IgG or with AAA-1 for 24 h, **a** AAA-1, but not control antibodies, dramatically increase the active form of SREBP-1 while SREBP-2 expression is not affected. *P*-value calculated with Mann–Whitney test. ** *P* = 0.008, ## *P* = 0.007; in **b** FASN and GPAT1 expression is strongly decreased after 24 h AAA-1 treatment. * *P* = 0.011, # *P* = 0.023, *** *P* = 0.001. In **c** Cells were treated with AAA-1 at different time points. * *P* = 0.029, *** *P* = 0.001, **** *P* < 0.001. One of three representative western blot is shown in each panel. Under each panel, respective data are expressed as scatter dot plot and median with range of band intensity volume/actin or tubulin intensity volume from three different experiments (n = 3). *P*-value calculated with Mann–Whitney test in **a** and **b** and with Kruskal–Wallis test in panel c). SREBP: Sterol regulatory element binding protein. FASN: Fatty acid synthetase. GPAT: Glycerol phosphate acyltransferase

As shown in Fig. 2a, 24 h of AAA-1 treatment increased the expression of SREBP-1, while the expression of SREBP-2 was unchanged, indicating that the triglyceride pathway is specifically upregulated in hepatocytes exposed to AAA-1. As both FASN and GPAT1 expressions were strongly reduced by AAA-1 (Fig. 2b, we evaluated the kinetics of SREBP-1, FASN and GPAT1 expression at different time points up to 24 h. Figure 2c reveals that AAA-1 cell stimulation led to a peak of SREBP-1 expression at 6–9 h, lasting up to 24 h, while the expression of FASN and GPAT1 peaked at 3–6 h before decreasing. These results are compatible with the existence of a negative feedback loop inactivating these enzymes in response to prolonged AAA-1-induced lipid overload over 24-h period.

### AAA-1 contribute to liver inflammation through TLR-2 and not through SREBP-1

Given the strong relationship between inflammation, initiation, and progression of NAFLD to NASH, we aimed to replicate the AAA-1 pro-inflammatory response previously observed in macrophages [12] in HepaRG cells.

**Fig. 3:**
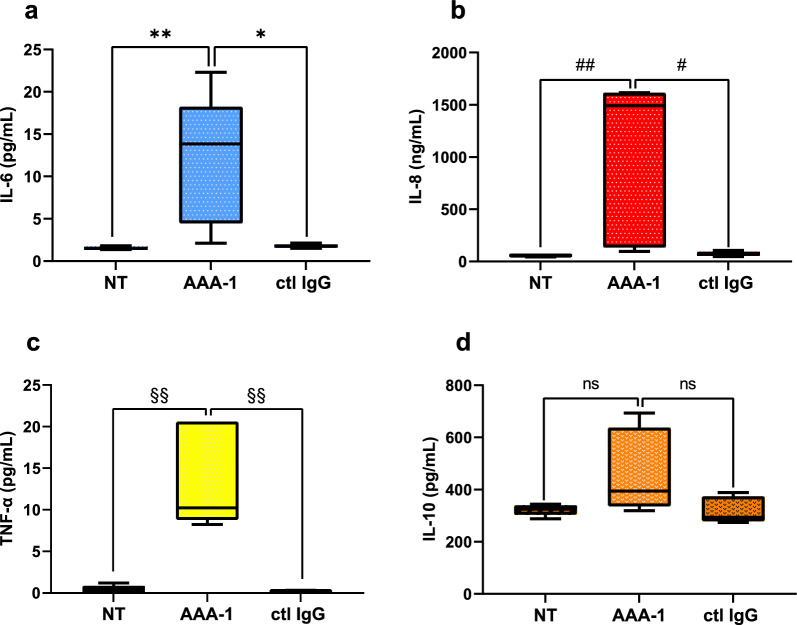
AAA-1 directly contribute to the pro-inflammatory response in hepatocytes. Levels of IL-6 (**a**), IL-8 (**b**), TNF-α (**c**) and IL-10 (**d**) were assessed in cell supernatant after treating HepaRG cells with AAA-1 or control IgG for 24 h. Results are expressed as median with IQR and range of five independent experiments (N = 5). **a** *P = 0.015, **P = 0.008; **b** ^#^*P* = 0.016, ^##^*P* = 0.008; **c** ^§§^*P* = 0.007 and **d** *P* = 0.095, by Mann–Whitney test

To do this, we analyzed supernatants obtained from HepaRG cells treated for 24 h with either AAA-1 or control IgG. We assessed the secretion of pro-inflammatory cytokines, specifically IL-6, IL-8, and TNF-α, while simultaneously measuring the presence of the anti-inflammatory cytokine IL-10 in order to understand the overall inflammatory status in relation to the function of AAA-1. As shown in Fig. 3, AAA-1 induced a significant increase in the secretion of IL-6 (panel a), IL-8 (panel b), and TNF-α (panel c), while there was no significant change in the production of IL-10 (panel d) when compared with the basal or control IgG conditions.

Since AAA-1 mediate foam cell formation through toll-like receptors (TLR)-2,4 and the canonical co-receptor CD-14 complex [12, 13, 17, 32], we examined whether similar TLR members were involved in this pro-inflammatory response observed in hepatic cells.

**Fig. 4:**
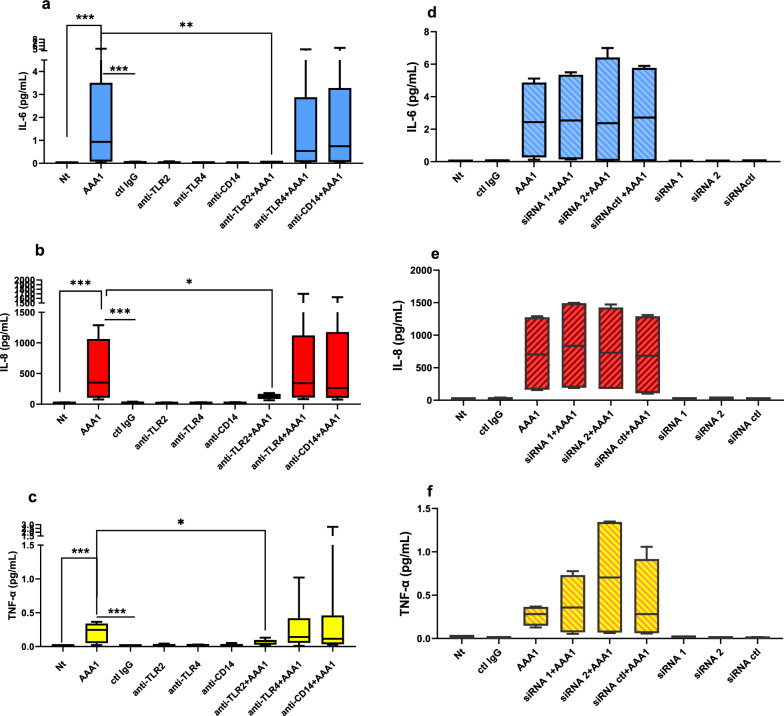
AAA-1 pro-inflammatory effects are TLR2-mediated. Levels of IL-6 (**a**), IL-8 (**b**) and TNF-α (**c**) are measured in the supernatant after cell treatment with AAA-1 alone or in combination with blocking antibodies to TLR-2, TLR-4, and CD-14. Results are expressed as median with IQR and range of six independent experiments (N = 6). Panel a) **P = 0.007, ***P = 0.001; panel b) *P = 0.038, ***P = 0.001; panel c) *P = 0.016, ***P = 0.001 by Mann–Whitney test. Levels of IL-6 (**d**), IL-8 (**e**) and TNF-α (**f**) are measured in the supernatant after cell treatment with AAA-1 alone or in combination with two different siRNAs targeting mSREBP1, or one siRNA used as control. Results are expressed as median with IQR and range of four independent experiments (N = 4)

When cells were co-treated with AAA-1 and blocking antibodies to TLR-2, 4 and CD-14, we observed a significant reduction of the inflammatory response only in presence of the blocking antibody to TLR-2 and not with blocking antibodies to CD-14 or TLR-4, which served as control antibodies, Fig. 4a–c. As TLR-4 was not found to be expressed in differentiated HepaRG cells (Additional file 1: Figure S1), these results point to a restricted TLR-2-mediated pro-inflammatory effect of AAA-1 on hepatocytes.

Because a recent study reports that SREBP-1 is necessary for the resolution of the TLR-mediated inflammation in macrophages [33], we further examined the possible contribution of SREBP-1 to the AAA-1 pro-inflammatory effects. Accordingly, we knocked-down SREBP-1 by transfecting HepaRG cells with two distinct validated siRNAs designed to target SREBP-1, and one siRNA as negative control. Gene silencing efficiency in HepaRG cells was evaluated through Western blot analysis, and the results are presented in Additional file 1: Figure S2. SREBP-1 silencing did not influence the TLR-2-mediated AAA-1 pro-inflammatory response, as indicated in Fig. 4d–f. These results may suggest that the AAA-1-mediated pro-inflammatory response is independent from hepatocyte fat accumulation induced by these autoantibodies.

### AAA-1 as an inducer of hepatocellular injury

**Fig. 5:**
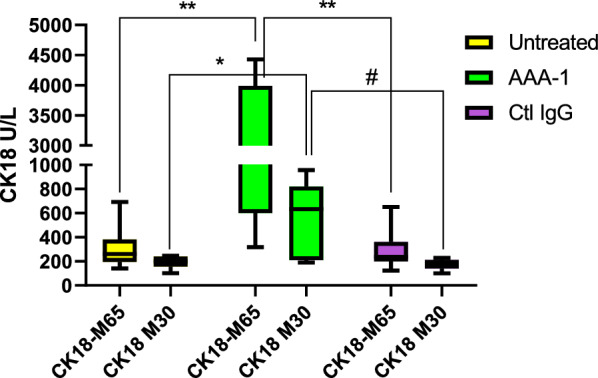
The impact of AAA-1 on hepatocellular injury. CK-18 secretion (M65 and M30 forms) has increased only in supernatants of AAA-1 treated cells. Results are expressed as median with IQR and range of five independent experiments (N = 5). *P = 0.041, #*P* = 0.015, **P = 0.004, ##*P* = 0.004 by Mann–Whitney test

Because non-alcoholic steatohepatitis (NASH), the progressive form of NAFLD is characterized by inflammation and cellular injury, we evaluated the impact of AAA-1 on the expression of the hepatocellular injury marker Cytokeratin-18 (CK-18). The level of both full-length CK-18 (M65) and caspase-cleaved CK-18 (M30) fragments in serum or plasma reflects the degree of necrotic hepatocellular injury and/or apoptosis [34]. We then measured it in the supernatants of HepaRG cells treated with AAA-1 or control IgGs. Both forms of CK-18 were evaluated: M65 that measures total cell death (necrosis and apoptosis) and M30 that measures only apoptosis [35]. Results shown in Fig. 5 indicated that both CK-18 forms were highly secreted in the cells supernatant following AAA-1 exposure, suggesting that AAA-1 may also be involved in liver injury, specifically during the stage immediately following inflammation.

### AAA-1 associate with biological and clinical factors in subjects with suspected NAFLD in the PREVEND general population cohort

To transpose our in vitro and animal data regarding the pro-steatotic effects of AAA-1 on hepatocytes, and to explore its relevance to CVD in humans, we analyzed the associations between AAA-1, NAFLD status (according to FLI), and CVD risk (according to 10-year FRS) [26] on 312 randomly selected PREVEND participants [22], and on the subgroup of individuals with a FLI ≥ 60, indicative of NAFLD [23–25].

The demographic and biochemical characteristics of the 312 PREVEND participants are presented in Additional file 1: Table S1. Prevalence of AAA-1 seropositivity was 32.3% and prevalence of a FLI ≥ 60 was 35.8% (Additional file 1: Table S1).

As shown in Additional file 1: Table S1, the FLI ≥ 60 subgroup, when compared to the FLI < 60 subgroup, had significantly elevated: diabetes prevalence, blood pressure, liver enzymes, glucose, triglycerides, FRS, and anthropometric indices, as well as lower HDL cholesterol levels. No difference in AAA-1 seropositivity and AAA-1 median levels were observed between these two groups. Spearman correlation analyses presented in Table 1 revealed a modest but significant positive association between AAA-1 and FRS (R = 0.11, *P* = 0.042), and a significant positive association between FLI and FRS (R = 0.49, *P* < 0.001). Linear regression analyses summarized in Table 2 confirmed those associations and indicated that both AAA-1 and FLI were independent predictors of FRS (β = 0.20, *P* = 0.034; β = 0.34, *P* < 0.001). These analyses also showed significant relationship between AAA-1 and alkaline phosphatase (ALP), (β = 0.16, *P* = 0.012) and between ALP and FRS (β = 0.97, *P* < 0.000), the latter remaining significant in multivariate linear regression analysis (β = 0.60, *P* < 0.001).

**Table 1:** Spearman’s rank correlation between levels of AAA-1, FLI, FRS and clinical characteristics of PREVEND participants

Variable	AAA-1		FLI		FRS	
	R	*P*	R	*P*	R	*P*
PREVEND participants (n = 312)						
Age	0.06	0.272	0.35	< 0.001	0.72	< 0.001
Waist circumference	0.004	0.931	0.91	< 0.001	0.42	< 0.001
Weight	0.007	0.893	0.79	< 0.001	0.19	0.001
BMI	0.03	0.223	0.84	< 0.001	0.38	< 0.001
Systolic blood pressure	0.02	0.745	0.47	< 0.001	0.61	< 0.001
Diastolic blood pressure	0.02	0.735	0.38	< 0.001	0.38	< 0.001
Total-c	− 0.03	0.502	0.09	0.102	0.22	< 0.001
LDL-c	− 0.03	0.552	0.06	0.271	0.21	0.001
HDL-c	− 0.09	0.103	− 0.5	< 0.001	− 0.30	< 0.001
TG	0.02	0.619	0.65	< 0.001	0.40	< 0.001
Plasma glucose	0.10	0.073	0.42	< 0.001	0.31	< 0.001
ALP	0.09	0.081	0.30	< 0.001	0.38	< 0.001
ALT	− 0.008	0.885	0.37	< 0.001	0.17	0.001
AST	0.10	0.051	0.28	< 0.001	0.21	0.001
GGT	0.07	0.211	0.67	< 0.001	0.33	< 0.001
AAA-1	–	–	0.04	0.461	0.11	0.041
FLI	0.04	0.464	–	–	0.49	< 0.001
FRS	0.11	0.042	0.49	< 0.001	–	–
FLI ≥ 60 PREVEND participants (n = 112)						
Age	0.002	0.982	0.17	0.065	0.64	< 0.001
Waist circumference	0.01	0.866	0.66	< 0.001	0.11	0.232
Weight	0.02	0.837	0.46	< 0.001	− 0.23	0.015
BMI	0.01	0.867	0.56	< 0.001	0.07	0.455
Systolic blood pressure	− 0.01	0.801	0.25	0.005	0.49	< 0.001
Diastolic blood pressure	− 0.03	0.711	0.11	0.252	0.08	0.394
Total-c	0.005	0.956	0.05	0.531	0.03	0.688
LDL-c	0.03	0.717	0.009	0.921	− 0.005	0.955
HDL-c	− 0.26	0.004	− 0.08	0.362	− 0.05	0.582
TG	0.09	0.308	0.28	0.002	0.26	0.004
Plasma glucose	0.1	0.233	0.13	0.175	0.35	0.001
ALP	0.3	0.003	0.14	0.137	0.17	0.072
ALT	0.02	0.813	0.24	0.012	0.11	0.231
AST	0.05	0.582	0.09	0.312	0.005	0.952
GGT	0.09	0.293	0.43	< 0.001	0.04	0.642
AAA-1	–	–	0.09	0.294	0.21	0.021
FLI	0.09	0.293	–	–	0.27	0.003
FRS	0.21	0.023	0.27	0.003	–	–
FLI < 60 PREVEND participants (n = 200)						
Age	0.08	0.262	0.27	< 0.001	0.70	< 0.001
Waist circumference	0.02	0.712	0.84	< 0.001	0.29	< 0.001
Weight	0.03	0.599	0.65	< 0.001	0.006	0.922
BMI	0.08	0.227	0.70	< 0.001	0.24	0.001
Systolic blood pressure	0.05	0.425	0.32	< 0.001	0.54	< 0.001
Diastolic blood pressure	0.06	0.385	0.30	< 0.001	0.39	< 0.001
Total-c	0.05	0.434	0.20	0.004	0.36	< 0.001
LDL-c	− 0.06	0.382	0.23	0.001	0.39	< 0.001
HDL-c	− 0.04	0.495	− 0.41	< 0.001	− 0.19	0.004
TG	0.01	0.853	0.5	< 0.001	0.23	0.001
Plasma glucose	0.07	0.265	0.22	0.001	0.13	0.050
ALP	0.02	0.723	0.31	< 0.001	0.41	< 0.001
ALT	− 0.009	0.891	0.22	0.001	0.04	0.514
AST	0.16	0.015	0.29	< 0.001	0.24	0.001
GGT	0.09	0.174	0.52	< 0.001	0.22	0.001
AAA-1	–	–	0.09	0.185	0.11	0.102
FLI	0.09	0.182	–	–	0.37	< 0.001
FRS	0.11	0.101	0.37	< 0.001	–	–

*Total-c* total cholesterol, *LDL-c* low-density lipoprotein cholesterol, *HDL-c* high-density lipoprotein cholesterol, *TG* triglycerides, *BMI* body mass index, *ALT* alanine transaminase, *AST* aspartate transaminase, *FLI* fatty liver index, *ALP* alkaline phosphatase, *GGT* gamma-glutamyl-transferase, *FRS* Framingham risk score, *AAA-1* anti-apolipoprotein A-1 IgG

**Table 2:** Linear regression analysis using FRS, AAA-1 or ALP as dependent variables in PREVEND participants

Variable	Univariate analysis		Multivariate analysis	
	β (95% CI)	*P-*value	β (95% CI)	*P*-value
PREVEND participants (n = 312)				
FRS				
AAA-1	0.30 (0.08–0.53)	0.007	0.20 (0.01–0.39)	0.034
ALP	0.97 (0.71–1.23)	< 0.001	0.60 (0.35–0.85)	< 0.001
FLI	0.41 (0.33–0.49)	< 0.001	0.34 (0.26–0.42)	< 0.001
AAA-1				
FLI	0.01 (− 0.02–0.06)	0.452	− 0.02 (− 0.07–0.02)	0.346
ALP	0.16 (0.02–0.30)	0.012	0.11 (− 0.03–0.26)	0.126
FRS	0.07 (0.02–0.13)	0.007	0.07 (0.006–0.13)	0.032
FLI				
AAA-1	0.1 (− 0.17–3.73)	0.452	− 0.11 (− 0.35–0.12)	0.345
ALP	0.98 (0.66–1.31)	< 0.001	0.44 (0.12–0.76)	0.006
FRS	0.61 (0.5–0.73)	0.001	0.55 (0.43–0.68)	< 0.001
ALP				
AAA-1	0.1 (0.01–0.19)	0.011	–	–
FLI ≥ 60 PREVEND participants (n = 112)				
FRS				
AAA-1	0.37 (0.008–0.74)	0.040	0.25 (− 0.11–0.63)	0.177
FLI	1.2 (0.42–2.08)	0.003	1.1 (0.28–1.94)	0.008
ALP	0.43 (0.02–0.83)	0.032	0.26 (− 0.15–0.67)	0.215
AAA-1				
FRS	0.09 (0.004–0.37)	0.041	0.06 (− 0.03–0.16)	0.172
FLI	0.20 (− 0.22–0.63)	0.346	0.02 (− 0.4–0.46)	0.891
ALP	0.34 (0.14–0.53)	0.001	0.30 (0.10–0.51)	0.003
FLI				
AAA-1	0.03 (− 0.04–0.12)	0.346	0.005 (− 0.07–0.09)	0.895
ALP	0.07 (− 0.01–0.15)	0.123	0.04 (− 0.04–0.13)	0.323
FRS	0.06 (0.02–0.1)	0.001	0.05 (0.01–0.09)	0.008
ALP				
AAA-1	0.28 (0.12–0.45)	0.001	–	–
FLI < 60 PREVEND participants (n = 200)				
FRS				
AAA-1	0.31 (0.06–0.57)	0.015	0.22 (− 0.002–0.44)	0.053
ALP	0.99 (0.68–1.31)	< 0.001	0.75 (0.44–1.06)	< 0.001
FLI	0.37 (0.25–0.48)	< 0.001	0.27 (0.15–0.39)	< 0.001
AAA-1				
FRS	0.09 (0.01–0.16)	0.018	0.08 (− 0.001–0.17)	0.051
FLI	0.04 (− 0.02–0.11)	0.187	0.01 (− 0.06–0.09)	0.681
ALP	0.10 (− 0.03–0.29)	0.271	0.06 (− 0.1–0.21)	0.955
FLI				
AAA-1	0.18 (− 0.08–0.46)	0.189	0.05 (− 0.2–0.31)	0.687
ALP	0.79 (0.43–1.14)	< 0.001	0.41 (0.04–0.78)	0.022
FRS	0.43 (0.29–0.57)	< 0.001	0.36 (0.2–0.51)	< 0.001
ALP				
AAA-1	0.05 (− 0.04–0.16)	0.201	–	–

Β unstandardized regression coefficient; CI, confidence interval; AAA-1, anti-apolipoprotein A-1; ALP, alkaline phosphatase; FRS, Framingham risk score; FLI, fatty liver index. Because the non-normal distribution of all variables, data were transformed in logarithmic value

Next, we considered only the 112 participants with FLI ≥ 60 (Table 3). AAA-1 seropositivity was 30.3% and was associated with higher median ALP, lower HDL cholesterol and tended to display higher FLI values compared to AAA-1 negative participants (*P* = 0.072). Furthermore, AAA-1 seropositive participants showed a higher 10-year FRS compared to seronegative participants (Table 3).

**Table 3:** Clinical characteristics of PREVEND participants with the FLI ≥ 60 according to AAA-1 status

	Overall (n = 112)	AAA-1 negative (n = 78)	AAA-1 positive (n = 34)	*P*-value
Demographic				
Age, yr	59 (50.5–69)	58.5 (50–70)	62 (52–67)	0.615
Males, no. (%)	43 (38.3)	29 (37.1)	14 (41.7)	0.428
Waist circumference, cm	107 (102–114)	106 (102–111)	107.7 (103–115.5)	0.188
Weight, kg	93 (84–102)	92.2 (83–101)	95.7 (86.5–102)	0.272
BMI, kg/m^2^	30.4 (28.8–32.9)	30.2 (28.7–32.4)	30.9 (28.8–33)	0.374
Systolic blood pressure mm Hg	134 (123–146)	133 (122–146)	140.5 (127.5–149)	0.352
Diastolic blood pressure mm Hg	76 (71–80)	76 (70–80)	75.5 (72–80)	0.752
Current smoker, no. (%)	22 (19.6)	12 (15.3)	10 (29.4)	0.156
Type 2 diabetes, no. (%)	16 (14.2)	10 (21.2)	6 (22)	0.577
FRS (%) ^b^	18.5 (11.7–30)	15.8 (11.1–28.8)	22.3 (15–30)	0.022
Biochemical				
Total-c mg/dl	189.8 (167.6–224.4)	189.8 (167.8–226.6)	190 (166.2–221.9)	0.912
LDL-c^a^ mg/dl	123.7 (100.2–155.8)	122 (100.2–158.4)	127 (98.4–155.8)	0.898
HDL-c mg/dl	32.4 (26.9–37.1)	34 (27.4–39.4)	28.6 (23.9–35.5)	0.032
TG mg/dl	162 (120.4–215.2)	161.6 (124.8–213)	170 (117.7–216.9)	0.755
Plasma glucose, mg/dl	90 (84.6–106.2)	90 (84.6–107.1)	91.8 (84.6–106.2)	0.832
ALP, U/l	47.5 (40–56.5)	45 (38–52)	52 (42–66)	0.008
ALT, U/l	7.9 (5.6–10.1)	8 (6.1–10.1)	7.7 (5.6–11)	0.919
AST, U/l	20 (16–26)	20 (16–24)	21.5 (14–27)	0.933
GGT, U/l	36 (24–54.5)	34 (22–51)	37.5 (28–60)	0.375
FLI^c^	82.3 (73.4–92.5)	81.4 (70.6–92.1)	87.6 (77.5–93.7)	0.072

All continuous variables are expressed as median (interquartile range [IQR]) or number [no.] (percentages [%]). *P*-value (Mann–Whitney U-test for continuous variables and Fisher's exact test for categorical variables)

*total-c* total cholesterol, *LDL-c* low density lipoprotein cholesterol, *HDL-c* high-density lipoprotein cholesterol, *TG* triglyceride, *BMI* body mass index, *ALT* alanine transaminase, *AST* aspartate transaminase, *FLI* fatty liver index, *ALP* alkaline phosphatase, *GGT* gamma-glutamyl-transferase, *FRS* Framingham risk score, *AAA-1* anti-apolipoprotein A-1 IgG

^a^LDL calculated according to Friedwald formula

^b^FRS: calculated based on: sex, age, smoking status, presence of diabetes, hypertension treatment, total cholesterol, HDL cholesterol

^c^FLI: (e 0.953*loge (triglycerides) + 0.139 * BMI + 0.718 * loge (ggt) + 0.053 * waist circumference − 15.745)/(1 + e 0.953 * loge (triglycerides) + 0.139 * BMI + 0.718 * loge (ggt) + 0.053 * waist circumference − 15.745) * 100

No association between AAA-1 seropositivity and other clinical or biochemical variables was observed. These observations were confirmed by Spearman correlation analyses (Table 1) where AAA-1 positively correlated with ALP and FRS, and inversely with HDL cholesterol. In addition, these associations were reproduced in univariate linear regression analyses (Table 2). While the association of FRS with FLI remained significant in multivariate analyses, the associations between AAA1 and FRS were lost when adjusting for FLI and ALP (Table 2).

These observations were not reproduced in participants with a FLI < 60 (Additional file 1: Table S2 and Table 1). However, AAA-1, FLI and ALP were still significantly associated with FRS (Table 2).

Considering that a 10-year FRS ≥ 20% is a robust CVD risk estimate [27], we performed logistic regression analyses in order to investigate a possible role of ALP, FLI and AAA-1 as predictors of FRS ≥ 20%. Table 4 indicated that in FLI ≥ 60 PREVEND participants only, AAA-1 seropositivity and continuous values thereof were associated with an increased risk of having a 10-year FRS ≥ 20% (OR: 2.44, CI 1.07–5.60, *P* = 0.032, and OR: 7.80, 95% CI 0.96–63.19, *P* = 0.051, respectively). These associations were not observed in all participants together, nor in FLI < 60 participants, and did not remain significant after adjustment for FLI and ALP; while both ALP and FLI independently predicted FRS ≥ 20% (Table 4). Taking together, these results indicate that FLI is independently associated with FRS, and that the previously reported associations between AAA-1 and FRS [16] are undermined by FLI.

**Table 4:** Logistic regression analysis using AAA-1, ALP or FLI as predictor of a high FRS

FRS ≥ 20%	Univariate analysis			Multivariate analysis			Multivariate analysis		
Predictor	OR	95% CI	*P-*value	OR	95% CI	*P-*value	OR	95% CI	*P*-value
PREVEND subjects (n = 312)									
AAA-1 positivity	0.79	0.47–1.33	0.395	1.03	0.57–1.85	0.922	–	–	–
AAA-1 continuous	2.11	0.84–5.30	0.113	–	–	–	1.85	0.64–5.3	0.252
ALP	1.04	1.02–1.06	< 0.001	1.03	1.01–1.05	0.001	1.03	1.01–1.05	0.001
FLI	1.02	1.01–1.03	< 0.001	1.02	1.01–1.03	< 0.001	1.02	1.01–1.03	< 0.001
FLI ≥ 60 PREVEND subjects (n = 112)									
AAA-1 positivity	2.44	1.07–5.60	0.032	1.70	0.69–4.21	0.235	–	–	–
AAA-1 continuous	7.80	0.96–63.19	0.051	–	–	–	3.90	0.41–37.8	0.235
ALP	1.03	1.0–1.05	0.022	1.02	0.99–1.05	0.117	1.02	0.99–1.05	0.135
FLI	1.06	1.02–1.1	0.001	1.05	1.01–1.09	0.004	1.05	1.01–1.09	0.003
FLI < 60 PREVEND subjects (n = 200)									
AAA-1 positivity	1.21	0.56–2.68	0.591	0.72	0.31–1.67	0.442	–	–	–
AAA-1 continuous	2.12	0.7–6.4	0.185	–	–	–	1.60	0.46–5.5	0.455
ALP	1.05	1.02–1.08	< 0.001	1.04	1.02–1.07	0.001	1.04	1.02–1.07	0.001
FLI	1.02	1–1.04	0.028	1.01	0.99–1.03	0.229	1.01	0.98–1.03	0.306

*FRS* Framingham risk score, *OR* odds ratio, *CI* confidence interval, *AAA-1* anti-apolipoprotein A-1 IgG, *ALP* alkaline phosphatase, *FLI* fatty liver index

In multivariate analysis, only the indicated predictors were included in the model

## Discussion

There are three key novel experimental findings of the current work. The first is that AAA-1 promote hepatocyte steatosis in vitro and in vivo, most likely by increasing lipogenesis due to the selective upregulation of SREBP-1 and the transient activation of two key enzymes involved in triglyceride synthesis (FASN and GPAT-1). Previous work demonstrated that AAA-1 could increase intracellular esterified lipid pools by increasing LDL uptake, stimulating the 3-hydroxy-3-methylglutaryl-CoA reductase (HMGCoR), the LDL receptor, and by decreasing passive diffusion in macrophages to generate foam cells, the hallmark of atherosclerosis [12, 14]. Because there is significant trafficking of both triglycerides and fatty acids into and out of the hepatocytes during feeding and fasting in order to maintain a steady state concentration of hepatic triglycerides [7], we cannot exclude that AAA-1 could exert an influence on their uptake or excretion. Considering that there are numerous transporter proteins and enzymes concomitantly involved in the fatty acid uptake and excretion/degradation in the liver [7], we therefore could not address this complex network in the current work. The second novel experimental finding is that AAA-1 elicit a TLR-2-dependent pro-inflammatory response by hepatocytes, independently of SREBP-1, while no effect was observed on the anti-inflammatory cytokine IL-10. The results indicate that AAA-1 generate inflammation mostly by stimulating the production of pro-inflammatory cytokines, without dampening the anti-inflammatory response, at least on IL-10 production known to display strong anti-inflammatory properties in the liver [36, 37]. These results concur and extend previous results on macrophages and neutrophils showing that AAA-1 pro-inflammatory effects are mediated by the TLR-2/TLR-4/CD-14 complex [13, 17, 32].

The third important finding of this study is that in top of eliciting a pro-inflammatory response in hepatocytes, our results indicate that AAA-1 also induce hepatocyte damage, as reflected by the production of CK-18, a cytoskeleton protein released upon hepatocytes ballooning, necrosis or apoptosis [38, 39]. Furthermore, because circulating CK-18 levels have been shown to be non-invasive biomarkers for NAFLD/NASH and liver fibrosis prediction [40, 41], knowing whether AAA-1 could elicit fibrosis is under active investigations. Taken together, these results raise the hypothesis that these antibodies could directly mediate the two necessary “hits” for NAFLD pathogenesis [42]. In this view, AAA-1 could participate in the first hit by inducing de novo lipogenesis dysregulation (accounting for 25–30% of the hepatic triglyceride content [30]). Secondly, by promoting inflammation and hepatocyte injury, these antibodies could also participate in the "second hit" of the hypothesis [42], probably contributing to the evolution of NAFLD to NASH, and even to fibrotic NASH. From a therapeutic perspective, if weight loss remains the cornerstone of NAFLD patient management, sodium–glucose co-transporter type-2 inhibitors (SGLT-2i) are emerging NAFLD therapeutic pharmacological molecules due to their ability to inhibit SREBP-1, hepatocyte inflammation, and liver fibrosis [43]. As AAA-1 have been shown to predict resistance to fat loss after dieting [44] and to predict reduced excess body mass index loss following bariatric surgery [45], and obviously act as antagonists of the beneficial properties of this class of pharmacological compounds, knowing if SGLT-2i could revert the pro-steatotic, and pro-inflammatory effects of AAA-1 is worth further study. Such investigations could validate or invalidate the hypothesis that AAA-1 assessment in NAFLD can be used to identify the subset of patients particularly likely to benefit from SGLT-2 inhibition.

We then aimed at translating this experimental evidence-driven hypothesis to humans by using a random subset of the general population-based cohort PREVEND where an elevated FLI was used as a proxy for NAFLD, as proposed by international guidelines [23, 25, 46]. Firstly, prevalence of elevated FLI and AAA-1 seropositivity were similar to what was previously reported in various population-based cohorts [16, 24, 25], and elevated FLI, which is known to predict CVD [47], was well independently associated with FRS, as shown before [48]. Secondly, the known AAA-1 associations with higher CV risk [16, 49, 50] were also reproduced, as well as the fact that NAFLD individuals (FLI > 60) had higher AST/ALT enzyme levels compared to non-NAFLD individuals. Taken together these findings suggest that the present random individual subset was well representative of the general population.

Consistent with the fact that CVD represents the major cause of death in NAFLD patients [51], the present general population study provides several innovative insights linking NAFLD, CVD and AAA-1.

Firstly, AAA-1 seropositivity was significantly associated with higher ALP levels in elevated FLI participants, and in the same group, AAA-1 positive subjects tended to have significantly higher FLI levels and lower HDL cholesterol compared to AAA-1 negative individuals. Despite being derived from a limited number of individuals, we consider the association between ALP and AAA-1 to be a relevant finding in our study because higher ALP is an independent predictor of FLI ≥ 60 mediating steatosis in vitro [52–54], is associated with NAFLD complications such as NASH and fibrosis [52, 54], and mediates vascular calcification [55, 56]. Whether AAA-1 could modulate ALP activity to induce steatosis or act separately is still an open question beyond the scope of the current work, especially as the exact mechanisms through which ALP influences steatosis are not known. Whether such ALP-related crosstalk could also explain the AAA-1 association with coronary artery calcification in obese individuals [57] remains to be established.

The key hypothesis-generating observation derived from the clinical part of the current translational study is that the association between AAA-1 and CVD may well be influenced by underlying hepatic steatosis. Our analyses indicated that the AAA-1 association with FRS ≥ 20% was lost as soon as FLI was included as a covariate. Although a power issue could explain such finding, this is the first report suggesting the existence of such association. If so, the independent nature of the link relating AAA-1 to CV hazards could be called into question, opening new research avenues bridging NAFLD and CVD.

Our study has several limitations. As we carried out a cross-sectional observational study in humans, a cause-effect relationship between AAA-1 and NAFLD cannot be ascertained. Importantly, although the PREVEND participants were representative, their number was rather modest, restricting the number of covariates to be used in adjusted models, and limiting result interpretation in the case of non-significant associations. The FLI is an accepted proxy for NAFLD in epidemiological studies [23–25]. As we could not calculate other non-invasive indices of liver fibrosis because of a lack of required biochemical characterization, and because liver biopsy-based diagnosis confirmation was not available, our clinical findings must be interpreted with caution and considered as preliminary.

Furthermore, because we focused ourselves on replicating mechanisms described previously in macrophages, we could not fully explore the SREBP-1-dependent mechanisms by which AAA-1 promote lipid droplet accumulation in hepatocytes and other pathological mechanisms cannot be excluded at this early stage of the investigations in this field. Moreover, suspecting lipofectamine (used as vector for SREBP-1 siRNA silencing) interference on bodipy staining imaging, we could not quantify the impact on lipid droplets with bodipy in our in vitro experiments, despite the concordant effects on all the relevant proteins and cytokines observed. Furthermore, based upon existing literature and because of prosaic considerations (animal tissue and cell supernatant availability), we focused on CK-18 as the sole surrogate marker of hepatocyte injury related to liver fibrosis in humans. Knowing whether AAA-1 could elicit fibrosis in an adapted cellular model such as hepatic stellate cells warrants further studies.

Finally, we did not assess autoantibodies directed against HDL cholesterol per se*,* nor other autoantibodies against other HDL proteins [58, 59]. Given the exploratory nature of this work, we deliberately focused on AAA-1 being: (i) the best characterized from analytical, clinical and experimental points of view [12, 13, 16–18, 21, 28, 50], and (ii) the unique HDL-related antibodies commercially available for in vitro and animal studies. Whether these findings could be replicated with other classes of anti-HDL antibodies remains to be shown.

## Conclusions

Although a single marker is likely to be insufficient to fully encompass the complexity of NAFLD pathogenesis, this translational study reveals that AAA-1 promote hepatocyte lipogenesis through SREBP-1 activation and foster inflammation and damage in hepatic cells, lending weight to the hypothesis that AAA-1 may be a pathogenic driver of NAFLD. Our results in humans with suspected NAFLD partly corroborate this hypothesis, highlighting complex interactions between FLI, ALP, AAA-1 and FRS, and even suggest that the previously reported link between AAA-1 and CVD could be influenced by the degree of liver steatosis. Future research should aim at assessing the accuracy of using AAA-1 to predict histology-based NAFLD/NASH/cirrhosis diagnoses, and at defining if AAA-1 could represent actionable therapeutic targets through SGLT-2 inhibition in order to promote the development of precision medicine in the field of NAFLD.

### Supplementary Information

**Additional file 1: Table S1.** Clinical characteristics of 312 PREVEND participants according to FLI status. **Table S2.** Clinical characteristics of PREVEND participants with the FLI < 60 according to AAA-1 status. **Figure S1.** TLR expression in HepaRG cells. **Figure S2**. Gene silencing efficiency in HepaRG. Additional Methods.

## Data Availability

The database of PREVEND will stay under supervision of the PREVEND board. The PREVEND dataset has been registered in the Maelstrom data catalogue: https://www.maelstrom-research.org/study/prevend which allows interested researchers to discover its existence and to apply for collaboration under the restriction that researchers from Groningen university retain the rights of intellectual input and property. Datasets will be shared after publication. Furthermore, data can be shared on request under the following conditions: A meaningful study question by the requester, Outline of the planned analyses, Valid methodology, Signed data sharing agreement that contains a confidentiality agreement, and includes an agreement on terms of collaboration.

## Abbreviations

NAFLD: Non-alcoholic associated fatty liver disease
NASH: Non-alcoholic steatohepatitis
CVD: Cardiovascular disease
AAA-1: Autoantibodies against apolipoprotein A-1
PREVEND: Prevention of Renal and Vascular Endstage Disease
FLI: Fatty Liver Index
FRS: Framingham Risk Score
T2D: Type 2 diabetes
FIB-4: Fibrosis-4
NFS: NAFLD fibrosis score
ELF: Enhanced Liver Fibrosis
FA: Fatty acid
IL-6: Interleukin 6
IL-8: Interleukin 8
TNF-α: Tumor Necrosis Factor α
TLR: Toll like receptor
LDL-c: Low-density lipoprotein cholesterol
HDL-c: High-density lipoprotein cholesterol
GGT: Gamma-glutamyltransferase
AST: Aspartate aminotransferase
ALT: Alanine aminotransferase
ALP: Alkaline phosphatase
SREBP: Sterol regulatory element binding protein
FASN: Fatty acid synthase
GPAT: Glycerol-3-phosphate acyltransferase
CK-18: Cytokeratin-18
SGLT2i: Sodium–glucose Cotransporter-2 Inhibitors
