# Supplementary material for: Antibody against apolipoprotein-A1, non-alcoholic fatty liver disease and cardiovascular risk: a translational study
Source: J Transl Med. 2023 Oct 5;21:694. doi: 10.1186/s12967-023-04569-7 (PMC10552329; doi:10.1186/s12967-023-04569-7)
Supplement: Supplementary file 1 — Additional file 1: Table S1. Clinical characteristics of 312 PREVEND participants according to FLI status. Table S2. Clinical characteristics of PREVEND participants with the FLI < 60 according to AAA-1 status. Figure S1. TLR expression in HepaRG cells. Figure S2. Gene silencing efficiency in HepaRG. Additional Methods. [file 12967_2023_4569_MOESM1_ESM.docx]

**ADDITIONAL MATERIAL**

**TABLES**

**Table S1. Clinical characteristics of 312 PREVEND participants according to FLI status.**

All continuous variables are expressed as median (interquartile range [IQR]) or number [no.] (percentages [%]). P-value (Mann-Whitney U-test for continuous variables and Fisher's exact test for categorical variables).Abbreviations: total-c: total cholesterol; LDL-c: low density lipoprotein cholesterol; HDL-c: high-density lipoprotein cholesterol; TG: triglyceride; BMI: body mass index; ALT: alanine transaminase; AST: aspartate transaminase; FLI: fatty liver index; ALP: alkaline phosphatase; GGT: gamma-glutamyl-transferase; FRS: Framingham risk score; AAA-1: anti-apolipoprotein A-1 IgG; OD: optical density

§LDL calculated according to Friedwald formula.

§§ FRS: calculated based on: sex, age, smoking status, presence of diabetes, hypertension treatment, total cholesterol, HDL cholesterol.

§§§ FLI: (e 0.953*loge (triglycerides) + 0.139*BMI + 0.718*loge (ggt) + 0.053*waist circumference - 15.745) / (1 + e 0.953*loge (triglycerides) + 0.139*BMI + 0.718*loge (ggt) + 0.053*waist circumference - 15.745) * 100

**Table S2. Clinical characteristics of PREVEND participants with the FLI < 60 according to AAA-1 status**

All continuous variables are expressed as median (interquartile range [IQR]) or number [no.] (percentages [%]). P-value (Mann-Whitney U-test for continuous variables and Fisher's exact test for categorical variables).

Abbreviations: total-c: total cholesterol; LDL-c: low density lipoprotein cholesterol; HDL-c: high-density lipoprotein cholesterol; TG: triglyceride; BMI: body mass index; ALT: alanine transaminase; AST: aspartate transaminase; FLI: fatty liver index; ALP: alkaline phosphatase; GGT: gamma-glutamyl-transferase; FRS: Framingham risk score; AAA-1: anti-apolipoprotein A-1 IgG;

§LDL calculated according to Friedwald formula.

§§ FRS: calculated based on: sex, age, smoking status, presence of diabetes, hypertension treatment, total cholesterol, HDL cholesterol.

§§§ FLI: (e 0.953*loge (triglycerides) + 0.139*BMI + 0.718*loge (ggt) + 0.053*waist circumference - 15.745) / (1 + e 0.953*loge (triglycerides) + 0.139*BMI + 0.718*loge (ggt) + 0.053*waist circumference - 15.745) * 100

**ADDITIONAL FIGURES**

**Figure S1**

**
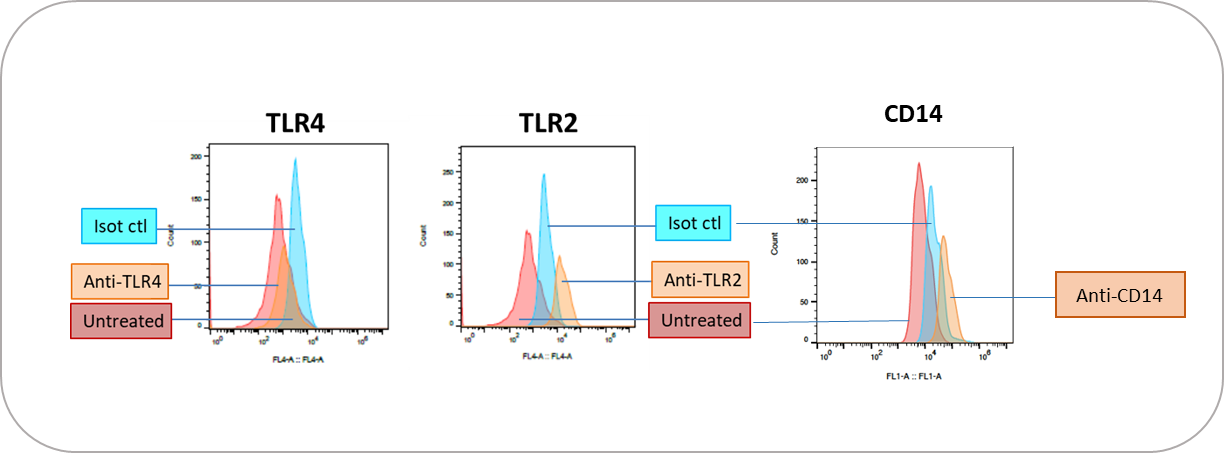
**

**Figure S1. TLR expression in HepaRG cells.** FACS analysis was performed to evalutate expression of TLR-2,TLR-4 and CD-14 in HepaRG cells. Cells were treated for 45 minutes with anti-TLR4, anti-TLR2 or anti-CD14 (show in orange). Cells were treated also with the specific IgG control (isot Ctl, shown in light blue) or with only culture medium (untreated shown in red). Results are representative of three independent experiments.

**Figure S2**


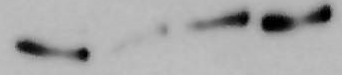

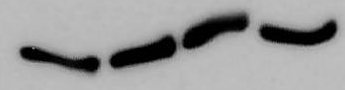


**Nt**

**siRNA_1**

**siRNA_2**

**siRNA_ctl**

**SREBP-1**

**Tubulin**

**Figure S2**. **Gene silencing efficiency in HepaRG.**

Gene silencing efficiency was analysed by western blot.

The intensity of SREBP-1 protein band decrease only in cells tranfected with siRNA targeting SREBP-1 gene. For negative control a scramble siRNA was used.

SREBP-1 protein analysisis was carried out at 24 hours post-transfection. α-tubulin was used as internal control.

**ADDITIONAL METHODS**

**Cells**

HepaRG cells obtained from Biopredic International (Saint Gregoire, France) and were cultured in Williams’ E medium (Gibco, Thermo Fisher Scientific, MA USA) supplemented with 10% (vol/vol) Cytiva Hy clone fetal clone II serum (U.S origin) (Cytiva, MA USA), 20 IU/mL penicillin, 20 µg/mL streptomycin from Gibco, 5 µg/mL bovine insulin solution (Sigma Aldrich, MO USA ) , 2 mM glutamine, and 50 µM hydrocortisone 21-hemisuccinate sodium salt (Sigma Aldrich). Cells were routinely passaged through plating them at low density (25 000 cells/cm2), allowing to reach 80% confluency after 3–5 days of culture, followed by culturing them for two weeks. Conventional dimethyl sulfoxide (DMSO)-mediated differentiation of HepaRG cells was performed through culturing cells for two weeks in the absence of DMSO, following by two additional weeks in the presence of 1.8% (vol/vol) DMSO (Sigma Aldrich), in order to promote expression of differentiated hepatic markers and functions, as previously described (1, 2).

**Oil Red O (ORO) staining for lipid droplet distribution**

ORO (Sigma-Aldrich), a fat-soluble dye, was used to detect neutral lipids (triglycerides, diacylglycerols, and cholesterol esters) in cryosectioned liver tissue. Briefly, sections after 10% formalin fixations were rinsed with water 2 for two minutes, then rinsed with 60% isopropanol and incubated 10 min with ORO at room temperature, after that time were rinsed again with 60% isopropanol and three times with water. Aquatex (Merck Millipore, MA USA) was used as a mounting media. Images were acquired with an automated upright slide scanning microscope in widefield, Zeiss Axio Scan.Z1 (Zeiss, Gottingen Germany). We used a 20X objective for all images. The images were collected using the Axio Scan.Z1 software (Zeiss). Quantification of the lipid ORO staining was obtained using Definiens Tissue Studio software (Definiens, Munich Germany). The score is based on the average number of pixels of red signal present in that specific region and tissue, as compared to the total area of the delineated region. This is defined as a percentage for each region analyzed.

**Bodipy staining for lipid droplet distribution**

Immunofluorescent detection of lipid droplet in HepaRG cells was done with the lipophilic dye Bodipy 493/503, 4,4-difluoro-3a,4a-diaza-s-indacene (Life Technologies, CA USA) while cell nuclei were stained with fluorescent stain Hoechst 33342 (Life Technologies).

Briefly, 12 000 differentiated HepaRG cells were seeded in a 96 well plate (Ibidi, Martinsried Germany) with Williams’ E medium supplemented as specified above. The next day, cells were fixed with 3% paraformaldehyde for 20 min. at room temperature. After cell washing, a 0.05% saponin (Sigma-Aldrich) solution containing Bodipy (1μg/mL) and Hoechst (5 μg/mL) was added and incubated at room temperature for 30 min shaking gently in the dark.

Images were acquired with the ImageXpress Micro Confocal® (Molecular Devices, CA USA)

using a 40X objective and quantified using MetaXpress Custom Module editor software.

After segmentation relevant masks were generated and then applied on the fluorescent images to extract relevant measurements. The average number of droplet per cell is reported on the graph.

**Protein purification and Western Blot analysis**

1.8 x106 differentiated HepaRG cells were plated onto a 6 well plate (Costar), the day after the cells were treated for 24 hours with polyclonal AAA-1 (Academy Bio-Medical Co, ref: 11AG2) or control IgG (Meridian Life Science, ref: A66200H) for 24 hours at 37°C both at the concentration of 40 μg/mL. After that the cells were lysed at 4°C for 20 minutes with radioimmunoprecipitation assay (RIPA) lysis buffer adding protease inhibitors (Complete tablets mini, Roche) and phosphatases inhibitors (Halt protease & phosphatase cocktail, Thermo Scientific) and then samples were centrifuged at 14.000 rpm at 4°C for 20 minutes. Protein concentration was determined by micro BCA Protein Assay Kit (Thermo Scientific).

Twenty micrograms of total protein extract were resolved by 8 or 10% polyacrylamide gel electrophoresis, under reducing conditions, and transferred to a polyvinylidene difluoride (PVDF) membrane (Immobilon, Millipore IPVH 00010) in presence of 20% methanol.

To reduce the possibility of non-specific binding the membrane was blocked by using 5% no-fat milk powder in tris-buffered saline and Tween 20 (T-TBS) for one hour at room temperature. After this step, the membrane was incubated under agitation with each primary antibody in 5% milk in T-TBS. The mouse anti-SREBP2 antibody (Cayman Chemicals, MI USA, ref: 10007663 ) was used 1:1000 overnight at 4 °C, the mouse anti-SREBP1 antibody (Novus Bio, CO USA, ref: NB600-58255) was used 1:1000 overnight at 4 °C, the rabbit anti-FASN antibody (Abcam, Cambridge UK, ref: Ab1223964) was used 1: 5000 overnight at 4 ° C, rabbit anti-GPAT1 antibody (Novus Bio, ref: NBP1-76907) was used 1: 1000 overnight at 4 ° C, finally mouse anti-Tubulin antibody (Santa Cruz Biotechnology, TX USA, ref: sc-23948) was used as control diluted 1:2000 1 hour at room temperature. As an alternative to anti-tubulin antibody, mouse anti-actin antibody diluted 1:5000 (Abcam, ref: ab8226) was also used as a control. Horseradish peroxidase–conjugated antisera from Dako (Agilent, CA USA) (1: 4000) were used to reveal primary binding, with detection by BM Chemiluminescence Blotting Substrate peroxidase (POD) from Roche Diagnostics (Mannheim, Germany).

Regarding the kinetics of protein expression we treated HepaRGs cells at different time points with AAA-1 (40 μg/mL) and processed for western blot analysis as described above.

**siRNA-mediated SREBP-1 gene knock-down**

siRNA transfections were performed with Lipofectamine RNAi-MAX (Invitrogen) in antibiotic-free medium according to manufacturer instructions.

A reverse transfection protocol was performed, using 96-well plates (Corning). Briefly, the transfection process started by adding 150 μl/well of each solution containing respectively one of the 3 siRNAs (siRNA1, siRNA2, siRNA ctrl): 3 μl siRNA (1μM) + 139.5 μl OPTI-MEM 1X (Gibco) and 7.5μl of Lipofectamine RNAiMAX diluted 1:10 in OPTI-MEM 1X.

Then 30 μl of OPTI-MEM 1X was added to each well. 30 min later, 1.5X104 per well of HepaRG cells were seeded using William's Medium E without antibiotics in a volume of 130 μl/well. 24 hours later, cells were treated with AAA-1 or control IgG for 24 hours.

The three validated siRNA against the SREBF1 target gene were from Ambion (Life technologies, Darmstadt, Germany).

siRNA1: part number 4390824, ID s129

siRNA2: part number 4392420, ID s131

siRNA3: negative control, 4390843

**AAA-1 assessment**

AAA-1 were measured as previously described (3-6). Briefly, Maxisorp plates (NuncTM, Roskilde, Denmark) were coated with purified, human-derived delipidated and unmodified apoA-1 (20 µg/mL; 50 µL/well) for 1 h at 37 °C. After being washed, all wells were blocked for 1 h with 2% BSA in a PBS at 37 °C. Participants’ samples were also added to a non-coated well to assess individual non-specific binding. After six washing cycles, a 50 µL/well of signal antibody (alkaline phosphatase-conjugated anti-human IgG; Sigma-Aldrich, St Louis, MO, USA, ref: A-3150), diluted 1:1000 in a PBS/BSA 2% solution, was added and incubated for 1 h at 37 C. After washing six more times, phosphatase substrate p-nitrophenyl-phosphate-disodium (Sigma-Aldrich, St Louis, MO, USA) dissolved in a diethanolamine buffer (pH 9.8) was added and incubated for 30 min at 37 °C (Molecular DevicesTM Filter Max F3, Molecular Devices, San Jose, CA, USA). OD450 was determined at 450 nm, and each sample was tested in duplicate. Corresponding non-specific binding was subtracted from the mean OD450 for each sample. The specificity of detection against lipid-free and unmodified apoA-1 has been previously determined by conventional saturation tests, Western blot, and LC-MS analyses (6) . The inter-assay coefficient of variation was 9% (n = 5), and the intra-assay CV was 5% (n = 5). For serum, the AAA1 seropositivity cut-off was previously specified and validated, and was set at an OD450 >0.64 for the 97.5th percentile of AAA-1 of healthy blood donors (3, 4, 6-9).

**Flow cytometry analysis**

Indirect immunofluorescence was performed to evaluate the expression of TLR-2, TLR-4 and CD-14 in HepaRG cells. Briefly, cells were seeded at a density of 300,000 cells per well in 96-well conical bottom plates, centrifugated for 5 minutes at 1000 rpm to carry out a first wash.

The cells were then incubated for 45 min- at 4 °C in the dark in 100 μl of Phosphate Buffered Saline (PBS)-1% BSA containing 2.5 μl of the antibody anti-TLR-2, anti-TLR4 or anti-CD14, labeled with APC probe (Biolegend, CA USA), or with 2.5 μl of the control isotype labeled with Allophycocyanin (APC) or Fluorescein isothiocyanate (FITC) probes.

The fluorescence intensity was analysed with the BD Accuri ™ C6 Plus Flow Cytometer and a minimum of 0.3 millions cells/sample was acquired. Analysis was preformed using FlowJo software (BD Biosciences, NJ, USA).

**Cytokeratin-18 assessment**

Supernatants derived from cells treated with AAA-1 or control IgG for 24 hours were analyzed for Cytokeratin-18 concentrations. The ELISA measurements of the Cytokeratin-18 concentrations were performed using the PEVIVA M65® ELISA and M30® ELISA kits (TECO medical AG, Switzerland), according to their corresponding protocols. Absorbance was measured with the FilterMax F3 Multi-Mode Microplate Reader, using the SoftMax Pro software, version 7.0.3.

**References**

1. Gripon P, Rumin S, Urban S, Le Seyec J, Glaise D, Cannie I, Guyomard C, Lucas J, Trepo C, Guguen-Guillouzo C. Infection of a human hepatoma cell line by hepatitis B virus. *Proc Natl Acad Sci U S A*. 2002;**99**(24):15655-15660.

2. Parent R, Marion MJ, Furio L, Trepo C, Petit MA. Origin and characterization of a human bipotent liver progenitor cell line. *Gastroenterology*. 2004;**126**(4):1147-1156.

3. Antiochos P, Marques-Vidal P, Virzi J, Pagano S, Satta N, Bastardot F, Hartley O, Montecucco F, Mach F, Waeber G, Vollenweider P, Vuilleumier N. Association between anti-apolipoprotein A-1 antibodies and cardiovascular disease in the general population. Results from the CoLaus study. *Thromb Haemost*. 2016;**116**(4):764-771.

4. Vuilleumier N, Pagano S, Combescure C, Gencer B, Virzi J, Raber L, Carballo D, Carballo S, Nanchen D, Rodondi N, Windecker S, Hazen SL, Wang Z, Li XS, von Eckardstein A, Matter CM, Luscher TF, Klingenberg R, Mach F. Non-Linear Relationship between Anti-Apolipoprotein A-1 IgGs and Cardiovascular Outcomes in Patients with Acute Coronary Syndromes. *J Clin Med*. 2019;**8**(7).

5. Vuilleumier N, Rossier MF, Pagano S, Python M, Charbonney E, Nkoulou R, James R, Reber G, Mach F, Roux-Lombard P. Anti-apolipoprotein A-1 IgG as an independent cardiovascular prognostic marker affecting basal heart rate in myocardial infarction. *Eur Heart J*. 2010;**31**(7):815-823.

6. Antiochos P, Marques-Vidal P, Virzi J, Pagano S, Satta N, Hartley O, Montecucco F, Mach F, Kutalik Z, Waeber G, Vollenweider P, Vuilleumier N. Impact of CD14 Polymorphisms on Anti-Apolipoprotein A-1 IgG-Related Coronary Artery Disease Prediction in the General Population. *Arterioscler Thromb Vasc Biol*. 2017;**37**(12):2342-2349.

7. Anderson JLC, Pagano S, Virzi J, Dullaart RPF, Annema W, Kuipers F, Bakker SJL, Vuilleumier N, Tietge UJF. Autoantibodies to Apolipoprotein A-1 as Independent Predictors of Cardiovascular Mortality in Renal Transplant Recipients. *J Clin Med*. 2019;**8**(7).

8. Antiochos P, Marques-Vidal P, Virzi J, Pagano S, Satta N, Hartley O, Montecucco F, Mach F, Kutalik Z, Waeber G, Vollenweider P, Vuilleumier N. Anti-Apolipoprotein A-1 IgG Predict All-Cause Mortality and Are Associated with Fc Receptor-Like 3 Polymorphisms. *Front Immunol*. 2017;**8**:437.

9. Frias MA, Virzi J, Batuca J, Pagano S, Satta N, Delgado Alves J, Vuilleumier N. ELISA methods comparison for the detection of auto-antibodies against apolipoprotein A1. *J Immunol Methods*. 2019;**469**:33-41.
